# Transcripts of the Prostate Cancer-Associated Gene *ANO7* Are Retained in the Nuclei of Prostatic Epithelial Cells

**DOI:** 10.3390/ijms24021052

**Published:** 2023-01-05

**Authors:** Olli Metsälä, Gudrun Wahlström, Pekka Taimen, Pirkko-Liisa Kellokumpu-Lehtinen, Johanna Schleutker

**Affiliations:** 1 Institute of Biomedicine, University of Turku, Kiinamyllynkatu 10, 25020 Turku, Finland; 2 FICAN West Cancer Center, University of Turku and Turku University Hospital, Kiinamyllynkatu 10, 25020 Turku, Finland; 3 Department of Pathology, Laboratory Division, Turku University Hospital, Kiinamyllynkatu 10, 25020 Turku, Finland; 4 Faculty of Medicine and Health Technology, Tampere University, Arvo Ylpön katu 34, 33520 Tampere, Finland; 5 Department of Genomics, Laboratory Division, Turku University Hospital, Kiinamyllynkatu 10, 25020 Turku, Finland

**Keywords:** prostate cancer, nuclear retention, mRNA, anoctamin

## Abstract

Prostate cancer affects millions of men globally. The prostate cancer-associated gene *ANO7* is downregulated in advanced prostate cancer, whereas benign tissue and low-grade cancer display varying expression levels. In this study, we assess the spatial correlation between *ANO7* mRNA and protein using fluorescent in situ hybridization and immunohistochemistry for the detection of mRNA and protein in parallel sections of tissue microarrays prepared from radical prostatectomy samples. We show that *ANO7* mRNA and protein expression correlate in prostate tissue. Furthermore, we show that *ANO7* mRNA is enriched in the nuclei of the luminal cells at 89% in benign ducts and low-grade cancer, and at 78% in high-grade cancer. The nuclear enrichment of *ANO7* mRNA was validated in prostate cancer cell lines 22Rv1 and MDA PCa 2b using droplet digital polymerase chain reaction (ddPCR) on RNA isolated from nuclear and cytoplasmic fractions of the cells. The nuclear enrichment of *ANO7* mRNA was compared to the nuclearly-enriched lncRNA *MALAT1*, confirming the surprisingly high nuclear retention of *ANO7* mRNA. *ANO7* has been suggested to be used as a diagnostic marker and a target for immunotherapy, but a full comprehension of its role in prostate cancer progression is currently lacking. Our results contribute to a better understanding of the dynamics of *ANO7* expression in prostatic tissue.

## 1. Introduction

Prostate cancer (PrCa) was the second most common cancer and ranked fifth among all cancer deaths worldwide in men in 2020. The incidence rates vary globally partly due to differences in diagnosis practices and access to high-level health care. The latter is also reflected in mortality rates. The most well-established risk factors for PrCa include advanced age, ethnic background, and a positive family history of the disease [1].

Although PrCa is a very common disease, its etiology remains largely unknown. Genetic factors play a major role in PrCa risk as 57% of the interindividual variation in risk is attributed to genetics [2]. The latest meta-analysis of genome-wide association studies increased the number of risk variants to 269, four of which are located in the *ANO7* gene [3,4]. The chromosomal location of *ANO7*, 2q37.2, was originally identified as a strong risk locus based on genetic linkage analyses of Finnish hereditary PrCa families [5].

*ANO7* (*TMEM16G, NGEP* (New Gene Expressed in Prostate)) belongs to the anoctamin family of membrane proteins (ANO1-10), which include Ca^2+^-activated ion channels and scramblases (reviewed in [6]). *ANO7* produces two high-confidence protein-coding transcripts: variant 1 and variant 2. Variant 1 encodes full-length ANO7 and is composed of exons 1 to 25. The truncated variant 2 lacks all transmembrane domains and is encoded by exons 1 to 4 [7,8]. The full-length ANO7 protein is mainly localized in the plasma membrane of prostatic epithelial cells (reviewed in [9]). ANO7 exhibits phospholipid scrambling activity [10] and minor ion channel activity [11,12]. However, the data remain inconclusive, and the function of ANO7 in prostate tissue is unknown. 

Studies have shown that *ANO7* mRNA expression decreases from low-grade to high-grade cancer [13,14] and that *ANO7* is downregulated in metastatic PrCa [15,16]. Previously, immunohistochemistry study results on the inverse relationship between protein expression and Gleason grade were inconclusive [17,18]. However, a recent analysis of greater than 13,000 prostate cancer patients showed that lower ANO7 protein expression in radical prostatectomy samples is associated with unfavorable clinicopathological parameters, including higher tumor stage, Gleason score, and preoperative prostate-specific antigen (PSA) level, as well as presence of lymph node metastasis, a positive surgical margin, and earlier biochemical recurrence [19].

Studies have reported a high variability in ANO7 protein expression in the prostate [18,19]. However, no attempts have been made to compare *ANO7* mRNA and protein levels in the same tissue sections. In one study, in situ hybridization using an *ANO7* probe was performed [7], but a comparison with protein expression was not conducted, apparently due to the lack of an anti-ANO7 antibody. According to that study, *ANO7* was expressed in basal and terminal epithelial (luminal) cells of the prostate. This is not consistent with a more recent RNA sequencing (RNA-Seq) study using sorted cells demonstrating that *ANO7* mRNA expression was specific to luminal cells [20].

In this work, we utilized a prostate tissue microarray (TMA) to detect both *ANO7* mRNA and protein expression in parallel sections of the same tissue. Our results support the downregulation of both *ANO7* mRNA and protein levels in high-grade prostate cancer, and *ANO7* mRNA expression appeared restricted to luminal cells. Surprisingly, a large fraction of *ANO7* mRNA is localized in the nucleus, indicating that regulation of ANO7 protein expression occurs at multiple levels.

## 2. Results

### 2.1. ANO7 Transcripts Are Expressed in Luminal Cells of the Prostatic Epithelium

To assess *ANO7* mRNA and protein in the parallel tissue sections, we utilized a tissue microarray containing samples from a clinically well-characterized set of patients [21]. ANO7 protein was detected using a commercial antibody that binds to the N-terminus of the protein. Thus, it also recognizes the putative short protein isoform of ANO7 encoded by transcript variant 2. For the detection of mRNA with fluorescent in situ hybridization (hereafter RNA-FISH), we utilized RNAscope, an in situ hybridization technique relying on multiple Z probes [22]. For a signal to be detected, two adjacent probes must bind to the target RNA, which reduces nonspecific off-target signals. The *ANO7* probes were designed to exclusively bind to the transcript encoding full-length *ANO7*.

**Figure 1:**
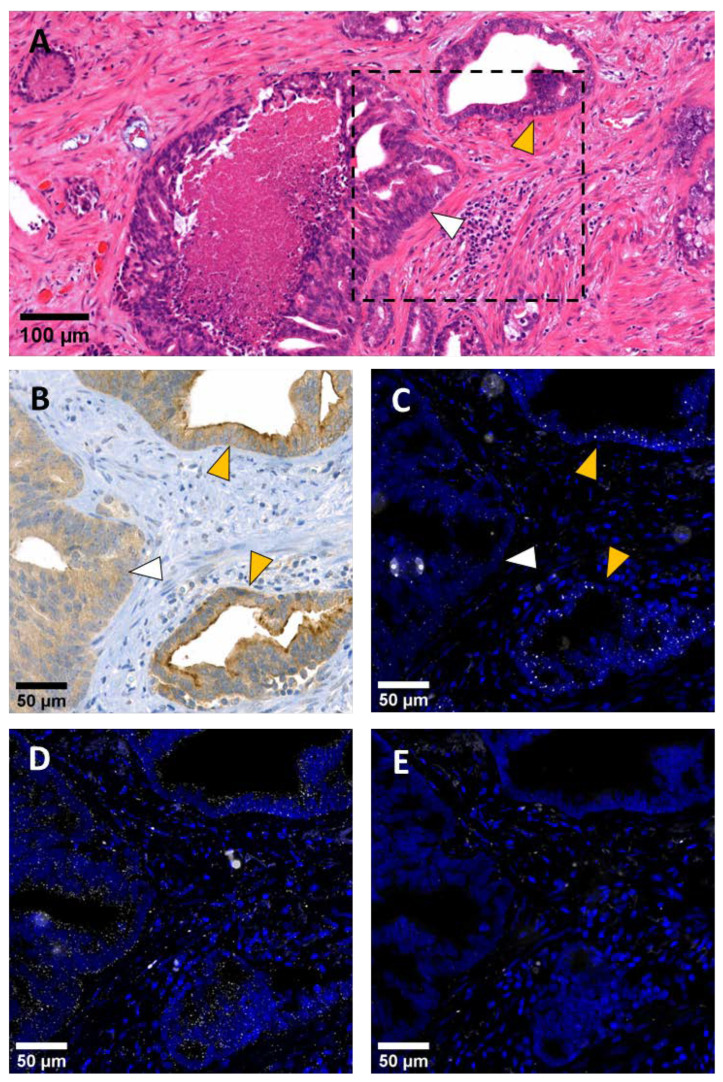
*ANO7* protein and mRNA expression correlate in the glandular structures of the prostate. Prostate tumor tissue sections containing well-differentiated glands (yellow arrowhead) and a region with high-grade cancerous growth (white arrowhead) stained with H&E (**A**), an anti-ANO7 antibody (**B**), or labelled with RNA-FISH for *ANO7* mRNA (**C**), positive control *PPIB* mRNA (**D**), or with a negative control probe against *dabB* (**E**). The probe signal in (**C**–**E**) is shown in white and the nuclear DAPI staining in blue. The *ANO7* mRNA signal (**C**) is prominent in the well-differentiated glands, which also show high anti-ANO7 staining intensity at the apical membrane (**B**). Markedly less *ANO7* mRNA signal is seen in the high-grade cancerous growth region (white arrowhead), which also displays loss of apical anti-ANO7 staining. The *PPIB* signal is present throughout the tissue with pronounced accumulation in epithelial cells (**D**). The negative control probe *dabB* reveals unspecific off-target signals (**E**).

As previously described [17,23], ANO7 protein was localized apically (lumen-facing side) in benign glands and low-grade (Gleason 3) cancerous acinar structures. In high-grade adenocarcinoma, ANO7 protein was downregulated or lost. Stromal cells did not express ANO7 protein (Figure 1A,B). Using RNA-FISH, we were able to detect tissue structures expressing *ANO7* mRNA. The *ANO7* mRNA signal was exclusively observed in the glandular epithelium, and no other structure showed distinct expression of *ANO7*. Additionally, the correlation of mRNA and protein in the high- and low-expressing regions was evident (Figure 1B,C). The housekeeping gene *PPIB*, which was used as a positive control, was expressed in both stromal and epithelial cells (Figure 1D). The negative control probe *dabB* exhibited similar off-target signals as described by Hilyard et al. [24] (Figure 1E).

**Figure 2:**
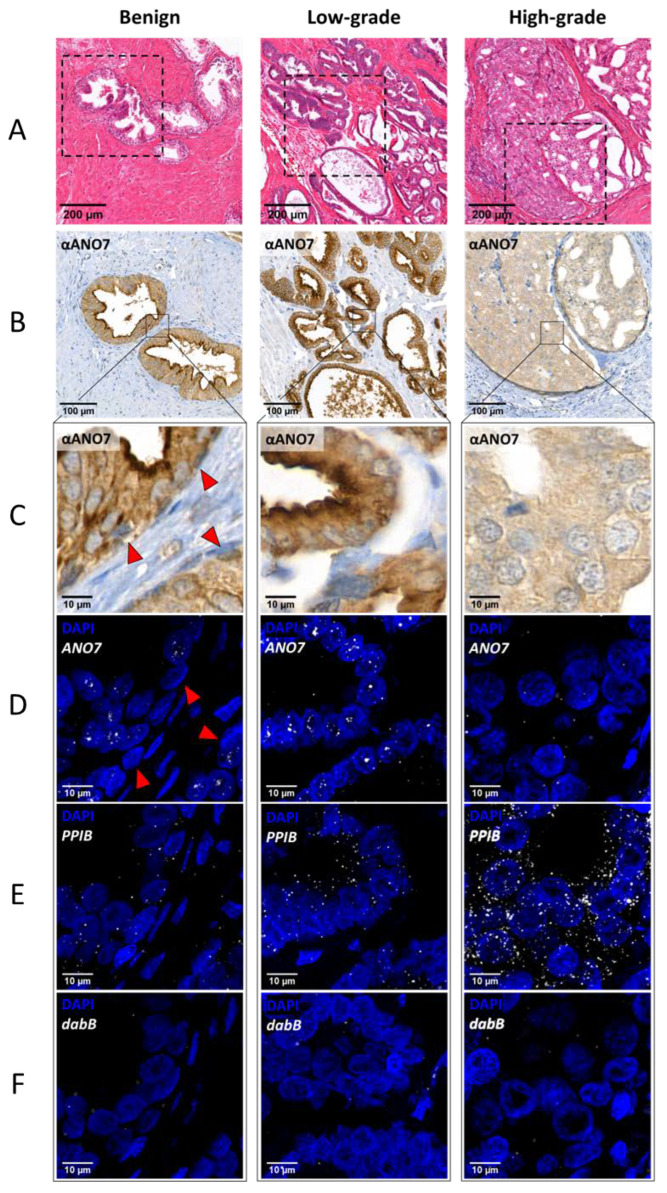
*ANO7* mRNA is expressed in luminal cells and accumulates in the nucleus. ANO7 protein and mRNA localization in benign glands (left column), low-grade cancer (middle column) and high-grade cancer (right column). (**A**) H&E staining of the tissue sections. (**B**,**C**) Anti-ANO7 staining of the boxed region indicated in (**A**) shows prominent staining at the apical surface of luminal cells in benign glands and low-grade cancer cells, whereas the staining is substantially reduced in high-grade cancer. (**D**–**F**) In the RNA-FISH images, the probe signal is shown in white for *ANO7* (**D**), *PPIB* (**E**), and *dabB* (**F**), and DAPI nuclear staining is shown in blue. (**D**) The *ANO7* mRNA signals display a similar nuclear clustering in benign glands and low-grade cancer, whereas in high-grade cancer, the signal is highly reduced. The red arrowheads in (**C**,**D**) indicate the epithelial basal cells, which are negative for *ANO7* expression. (**E**) The *PPIB* spots are widely scattered and increase in number with cancer grade, in contrast to *ANO7*.

Figure 2 shows representative images of ANO7 protein localization in benign glands, low-grade cancer, and high-grade cancer (Figure 2A–C) and RNA-FISH mRNA signal patterns for *ANO7* (Figure 2D), *PPIB* (Figure 2E), and *dabB* (Figure 2F) from the corresponding regions. In the benign glands, the *ANO7* protein and mRNA are located in the inner cell layer lining the acini (Figure 2C,D), which corresponds to the luminal cells [25]. In low-grade cancer, the *ANO7* signal pattern was very similar to that observed in benign glands (Figure 2D) and no *ANO7* mRNA was detected in the basal cells (Figure 2D, red arrowheads). In contrast to *ANO7*, *PPIB* expression gradually increased from benign to high-grade cancer (Figure 2E).

### 2.2. ANO7 Transcripts Are Enriched in the Nucleus of Benign Prostate Epithelial Cells and Cancer Cells

In the RNA-FISH images, the signal for *ANO7* mRNA in benign glands and low-grade cancer appeared as small point-formed foci and dense clusters of a few individual foci; the latter always colocalized with the nuclear staining (Figure 2D). These findings contrast with those noted for the positive control gene *PPIB*, the signal of which was distributed more evenly between the nucleus and the cytoplasm (Figure 2E). To study the mRNA distribution between the nucleus and cytoplasm, confocal images of 14 regions of interest (ROIs) from seven individual subjects and greater than 1400 cells (>600 from benign glands and low-grade cancer and >800 from high-grade cancer) for *ANO7* and *PPIB* were analyzed to assess the nuclear to cytoplasmic ratio of the transcripts. Due to the similarity of *ANO7* expression in benign glands and low-grade cancer, these samples were combined and compared against high-grade cancer. The number of segmented signal spots was counted from the image stack reconstituted into 3D images, and the percentage of nuclear transcripts was calculated from the total number of transcripts.

**Figure 3:**
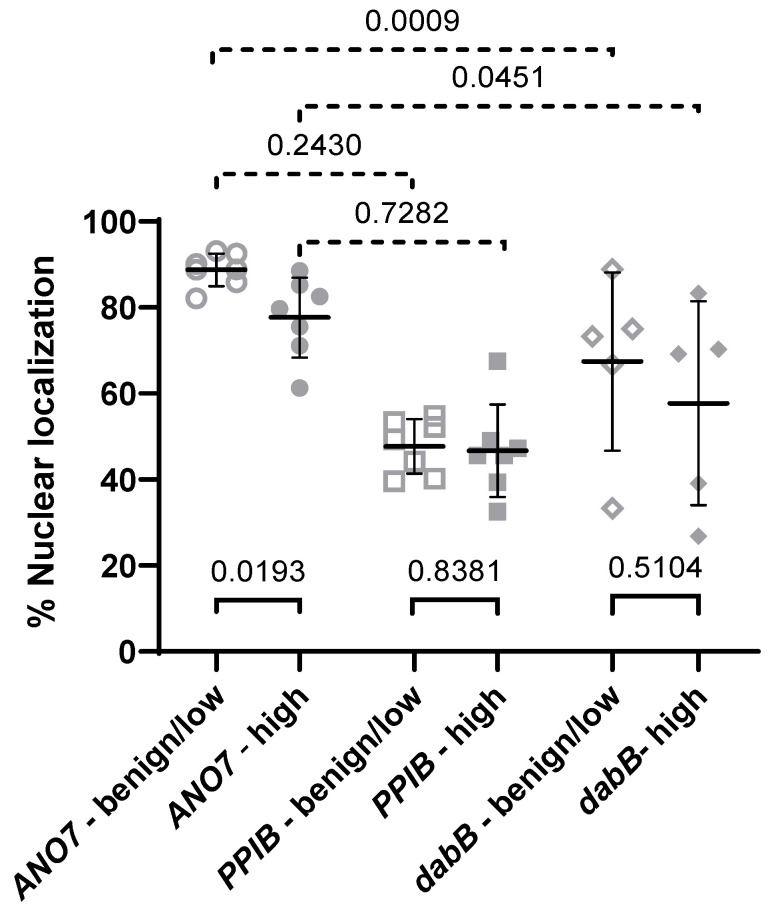
Quantification of the nuclear enrichment of *ANO7* transcripts in prostate tissue. The scatter dot plot shows the percentage nuclear localization of transcripts analyzed from the prostatic tissue RNA-FISH confocal images. The mean values and standard deviations are indicated. The *ANO7* transcripts are enriched in the nucleus in both tissue categories, and there is a statistically significant shift towards cytoplasmic localization in high-grade cancer compared to benign glands and low-grade cancer (*p* = 0.0193). Transcripts from the positive control gene *PPIB* transcripts are distributed equally between the nucleus and cytoplasm, and there is no difference between the tissue categories (*p* = 0.8381). The distribution of the off-target signals from the negative control probe *dabB* is wide, and there is no difference between the tissue categories (*p* = 0.5104). The variances were statistically unequal between *ANO7* and the negative control *dabB* (*p* = 0.0009 in benign/low and 0.0451 in high), but not between *ANO7* and the positive control *PPIB* (*p* = 0.2430 in benign/low and 0.7282 in high).

The results of the nuclear localization analysis are presented in Table 1 and Figure 3. The mean nuclear proportion of *ANO7* transcripts was 88.8% in benign and low-grade cancer and 77.7% in high-grade cancer, and the difference was statistically significant (*p* = 0.0193). The *PPIB* nuclear to cytoplasm ratio remained unchanged at 47.7% and 46.7% in benign to low-grade and high-grade cancer, respectively (*p* = 0.8381). The nuclear fraction of the off-target *dabB* signals was highly variable between the ROIs. As noted in Figure 2D and F, the off-target signal pattern in the negative control showed some similarity to the low signal of the *ANO7* probe in high-grade cancer. Therefore, we tested the equality of variance of the signal distribution in the ROIs for *ANO7* vs. *dabB* and *ANO7* vs. *PPIB,* which was significantly different in the former (*p* = 0.0009 in benign/low-grade cancer and *p* = 0.0451 in high-grade cancer) but not in the latter (*p* = 0.2430 in benign/low-grade cancer and *p* = 0.7282 in high-grade cancer). This indicates specificity of the *ANO7* signal. These results show that a substantial fraction of *ANO7* transcripts is retained in the nucleus throughout cancer progression in primary tumors. The reduction in the fraction of nuclear transcripts in high-grade cancer suggests that along with the downregulation of *ANO7* gene expression, nuclear retention is also attenuated.

### 2.3. ANO7 Transcripts and the Nucleus-Enriched MALAT1 Are Distributed Similarly between Nuclear and Cytoplasmic Compartments in Prostate Cancer Cell Lines

**Figure 4:**
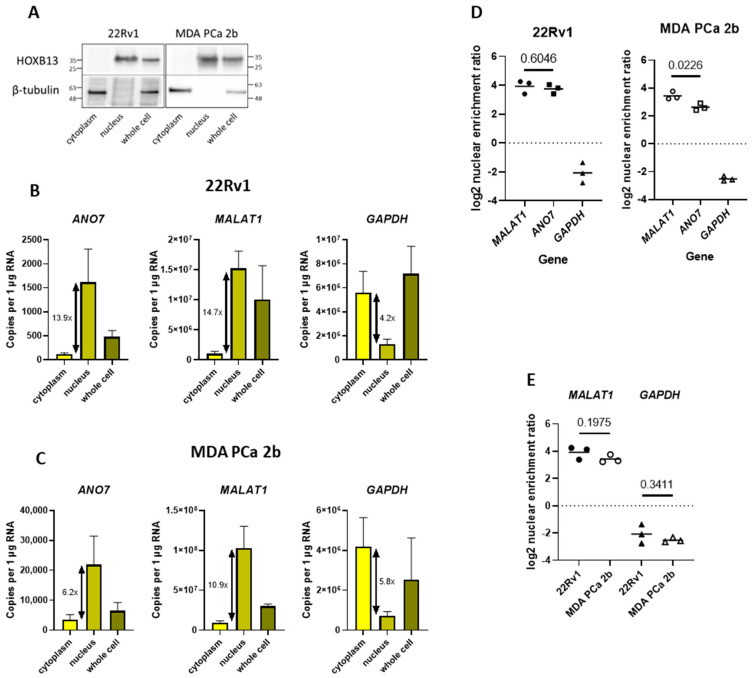
*ANO7* mRNA is enriched in the nuclear fraction of prostate cancer cell lines MDA PCa 2b and 22Rv1. (**A**) The purity of the fractions was verified at the protein level using Western blotting, showing that HOXB13 was exclusively detected in the nuclear fraction and β-tubulin in the cytoplasmic fraction. (**B**,**C**) Copies per 1 ug of RNA of *ANO7*, *MALAT1* and *GAPDH* in 22Rv1 (**B**) and MDA PCa 2b (**C**) measured with ddPCR. *ANO7* and *MALAT1* are enriched in the nuclear fraction, and *GAPDH* is enriched in the cytoplasmic fraction. The fold difference between the cytoplasmic and nuclear copy number is indicated next to the bars. (**D**) A comparison of the log2 nuclear enrichment ratios indicates a statistically significant difference between *ANO7* (log2 ratio 2.6) and *MALAT1* (log2 ratio 3.4) in MDA PCa 2b cells (*p* = 0.0226), but not in 22Rv1 cells (*ANO7* log2 ratio 3.7 and *MALAT1* log2 ratio 3.9; *p* = 0.6046). (**E**) A comparison of *MALAT1* and *GAPDH* log2 nuclear enrichment ratios between the cell lines does not reveal a significant difference.

The enrichment of *ANO7* transcripts in the nucleus was validated using *ANO7*-expressing prostate cancer cell lines MDA PCa 2b and 22Rv1. Total RNA was isolated from a whole-cell lysate and nuclear and cytoplasmic cellular fractions, which were verified for purity at the protein level using Western blotting. The cytoplasmic marker protein β-tubulin, which is a component of the cytoskeleton, was exclusively detected in the cytoplasmic fraction, and a nuclear marker protein, the transcription factor HOXB13, was exclusively detected in the nuclear fraction (Figure 4A). The absolute transcript copy number in total, cytoplasmic, and nuclear RNA was measured by droplet digital polymerase chain reaction (ddPCR), and the transcript copy numbers were normalized to 1 µg of RNA within each fraction. The long noncoding RNA (lncRNA) gene *MALAT1* was used as a control for transcripts enriched in the nucleus [26], and the protein-coding gene *GAPDH* was used as a control for transcripts enriched in the cytoplasm [27]. The mean transcript copy numbers per 1 µg of RNA for each fraction are presented in Table 2. As expected, *MALAT1* transcripts were highly enriched in the nucleus. The transcript copy number of the detected nuclear *MALAT1* was 14.7-fold higher than that of the cytoplasmic transcripts in 22Rv1 cells and 10.9-fold higher in MDA PCa 2b cells. In contrast, protein-coding *GAPDH* transcripts were enriched in the cytoplasmic fraction. The transcript copy number of cytoplasmic *GAPDH* was 4.2-fold higher in 22Rv1 cells and 5.8-fold higher in MDA PCa 2b cells compared with that noted in the nuclear fraction. Analysis of the *ANO7* transcript distribution revealed a pattern very similar to MALAT1. The transcript copy number of the detected nuclear *ANO7* was 13.9-fold higher than that of the cytoplasmic transcripts in 22Rv1 cells and 6.2-fold higher in MDA PCa 2b cells (Figure 4B,C). For statistical calculations, log2 enrichment values were calculated. A positive value corresponds to nuclear enrichment, and a negative value corresponds to cytoplasmic enrichment. *ANO7* was enriched in the nucleus with a mean log2 ratio of 3.75 and 2.63 in 22Rv1 and MDA PCa 2b cells, respectively, and a comparison against *MALAT1* showed a significant difference in MDA PCa 2b cells (*p* = 0.0226) but not in 22Rv1 cells (*p* = 0.6046) (Figure 4D). The control genes showed a uniform distribution in the cell lines with log2 nuclear enrichment ratios of 3.9 and 3.4 (*p* = 0.1975) for *MALAT1* and log2 cytoplasmic enrichment ratios of −2.1 and −2.5 (*p* = 0.3411) for *GAPDH* in 22Rv1 and MDA PCa 2b cells, respectively (Figure 4E). Thus, it can be concluded that the nuclear enrichment of *ANO7* in both cell lines confirms our findings in the RNA-FISH nuclear enrichment analysis.

## 3. Discussion

In this work, we show that the major transcript of *ANO7*, namely, variant 1 encoding the full-length protein, is enriched in the nucleus throughout PrCa progression. We investigated the subcellular localization of the transcripts in benign prostatic glands and low-grade and high-grade prostate cancer and validated the results in cancer cell lines. In the TMAs, the changes in RNA-FISH and immunohistochemistry signal intensities correlated spatially in the tissue, suggesting that ANO7 mRNA and protein expression levels are correlated. To the best of our knowledge, this is the first study in which both ANO7 mRNA and protein have been analyzed in parallel tissue sections of the same prostatic region.

Our observations suggest that in the normal prostatic epithelium, *ANO7* is expressed in luminal cells but not in basal cells, as the RNA-FISH signal was not detected in the basal layer of the epithelium (Figure 2D). Anti-ANO7 staining in both basal and luminal cells has been reported [19,28]. The antibody used in both of these studies, however, is not completely specific to ANO7 (https://v18.proteinatlas.org/ENSG00000146205-ANO7/antibody (accessed on 7 December 2022)), and the signal in basal cells was also observed in patients homozygous for a mutation that causes a severe splicing defect in *ANO7* [28]. Bera et al. [7] concluded from in situ hybridization data that *ANO7* mRNA is expressed in basal and terminal epithelial cells of the epithelium. This finding conflicts with the sorted cell RNA-seq data by Henry et al. [20], which indicated that *ANO7* is specific to luminal cells of the epithelium. Based on these data, whether *ANO7* is expressed in both basal and luminal cells or exclusively in luminal cells remains an open question.

The results presented here regarding the localization of the transcripts show that greater than 70% of the *ANO7* transcripts are localized in the nucleus of *ANO7*-expressing cells. The *ANO7* mRNA clusters appear as large high-intensity dots in the RNA-FISH (Figure 2D). This could potentially affect the segmentation of the signal spots, reducing the number of counted transcripts. However, this error would only underestimate the nuclear enrichment. Thus, *ANO7* transcripts are enriched at least to the level indicated here. In high-grade cancer, in which ANO7 is expressed at a very low level, the transcript distribution might be affected by possible off-target signals; we did indeed observe a greater variation in signal localization in high-grade cancer compared to low-grade cancer (Figure 3). Nonetheless, *ANO7* nuclear enrichment was consistently high in all analyzed ROIs in high-grade cancer, whereas the negative control *dabB* showed high variability. Therefore, it could be concluded that *ANO7* mRNA is retained in the nucleus both in benign tissue and in cancer. The subtly reduced nuclear retention in high-grade cancer will not have a significant impact on the amount of translated protein given that the overall gene expression level is considerably reduced, as also revealed by the very low protein expression in high-grade cancer (Figure 2B,C).

Our finding was validated in the PrCa-derived cell lines MDA PCa 2b and 22Rv1. The distribution of *ANO7* mRNA between the nucleus and the cytoplasm was compared to the nuclearly-localized lncRNA *MALAT1* and predominantly cytoplasmic *GAPDH* mRNA. *ANO7* mRNA was enriched in the nuclear compartment, similar to that noted for *MALAT1*, whereas *GAPDH* was enriched in the cytoplasmic fraction (Figure 4). These cell lines are derived from a high-grade tumor xenograft (22Rv1) [29] and a metastasis (MDA PCa 2b) [30]. Thus, they are representative of high-grade cancer tissue sections. We are not aware of a cell line derived from normal prostate that would express *ANO7*, therefore, we have not been able to validate the results obtained from the benign prostate tissue in situ hybridization. We have recently shown that the *ANO7* PrCa risk SNP rs77559646, which is located in the intron 4 splice donor region, causes a severe splicing defect. Nevertheless, equal levels of *ANO7* mRNA appeared to be expressed from both copies of the gene in prostate tissue [28]. We therefore hypothesized that a large fraction of *ANO7* mRNA might be nuclearly retained without being degraded, given that an aberrantly spliced RNA with in-frame premature termination codons that has been exported to the cytoplasm should be targeted for degradation [31]. The results presented here confirm that nuclear retention of *ANO7* indeed occurs. Interestingly, an analysis of mRNAs enriched in either the nuclear or the cytoplasmic fraction of brain tissue identified *ANO7* as one of the genes displaying nuclear enrichment [32]. Several classes of noncoding RNAs are localized to and functional in the nucleus. More recently, it has been recognized that some mRNAs are also enriched in the nucleus (reviewed in [33]). Nuclear retention can have many functional properties, such as reducing cytoplasmic gene expression noise [34]. In addition, retained intron-containing mRNA can act as a nuclear reservoir for the rapid production of mature mRNA upon external signaling or stress, and the ratio between intron-containing and fully-spliced mRNA is in some cases affected by the developmental stage of cells (reviewed in [35]).

Palazzo and Lee [36] reviewed the mechanisms of nuclear retention and stated that seemingly any stable long RNA is potentially exported from the nucleus, unless it contains elements mediating nuclear retention. Intron retention is a known mechanism behind the nuclear accumulation of transcripts [37,38,39]. We have shown that some introns in *ANO7* are partially retained in patient RNA-Seq data [28]. Thus, we hypothesize that the retained introns in *ANO7* may harbor nuclear retention elements. However, the mechanism that mediates the nuclear retention of *ANO7* as well as its functional importance remains to be discovered.

*ANO7* has been suggested to be used as a prognostic marker for PrCa [18,19] and as a target for PrCa immunotherapy [40]. The data we present in this paper help to understand the relationship between *ANO7* mRNA and protein expression. The strong association of *ANO7* and PrCa makes the gene very interesting in the context of cancer progression, whereas the reduced expression in advanced and metastatic cancer may indicate that the gene has lost its relevance in cellular functions in advanced cancer. Therefore, future studies aiming at elucidating the role of ANO7 should focus on benign tissue and low-grade cancer.

## 4. Materials and Methods

### 4.1. Patient Samples

A subset of formalin-fixed and paraffin-embedded (FFPE) tissue samples was used from Finnish patients in the PROSTY study [21]. TMAs were prepared from the original FFPE blocks, and the TMAs were sectioned for hematoxylin and eosin (H&E) staining, immunohistochemistry, and fluorescent in situ hybridization. Representing areas of benign gland, low-grade cancer, and high-grade cancer were selected for analysis. Gleason pattern 3 represents low-grade cancer, and Gleason patterns 4 and 5 represent high-grade cancer. This research was approved by the Institutional Review Boards of the Tampere University Hospital, and all patients provided written informed consent.

### 4.2. Fluorescent In Situ Hybridization

An RNAScope Multiplex Fluorescent v2 kit (323110, Advanced Cell Diagnostics, Newark, CA, USA) was used for fluorescent in situ hybridization. The 20ZZ probe against *ANO7* targeted bases 1015–1979 (NCBI reference sequence NM_001001891.3), the 16ZZ positive control probe targeted bases 139–989 in *PPIB* (NM_000924.4), and the 10ZZ negative control probe targeted bases 414–862 in the *B. subtilis* gene *dapB* (EF191515). The probes were labeled with Opal 570 fluorophore (FP1488001KT, Akoya Biosciences, Menlo Park, CA, USA) diluted 1:1500. The sections were mounted in ProLong™ Diamond Antifade Mountant with DAPI (P36962, Thermo Fischer Scientific, Waltham, MA, USA). The protocol was performed according to the manufacturer’s recommendations for prostatic tissue FFPE samples.

### 4.3. Immunohistochemistry

The immunohistochemistry for the TMA sections was performed as described [28]. The primary antibody against ANO7 was HPA035730 (Sigma-Aldrich, St. Louis, MO, USA) diluted 1:200.

### 4.4. Microscopy

The RNA-FISH TMAs were scanned with a Pannoramic MIDI slide scanner (3DHISTECH, Budapest, Hungary) equipped with a PCO edge 4.2 camera (Excelitas PCO, Kelheim, Germany), a 20× Plan-Apochromat objective, and filters for DAPI (445/50 nm) and Opal 570 (605/70 nm). The exposure times for the probe channel (Opal 570) were 300 milliseconds (ms) for *ANO7* and 600 ms for *PPIB* and *dabB*. The H&E and immunohistochemistry slides were scanned with a Pannoramic 1000 slide scanner (3DHISTECH, Budapest, Hungary) equipped with an Q-12A-180Fc camera (Adimec, Eindhoven, The Netherlands) and a Plan-Apochromat 20× objective, and images were captured using CaseViewer software version 2.4 (3DHISTECH, Budapest, Hungary).

The ROIs for analyzing *ANO7* transcripts were selected from the scanned images based on the following criteria: definable and intact tissue structures in the H&E-stained sections, detectable signal and consistent pattern in the positive control, and no excessive signal in the negative control.

The images for intracellular transcript localization analysis were acquired with a CSU-W1 spinning disc confocal microscope (3i, Denver, CO, USA) equipped with a Prime BSI sCMOS camera (Teledyne Photometrics, Tucson, AZ, USA), a 63× Plan-Apochromat oil-immersion objective (Zeiss, Oberkochen, Germany), and DAPI (445/45 nm) and Opal 570 (617/73 nm) filters. The exposure time varied according to the punctual maximal signal intensity representing individual transcripts, as the aim was to only detect the ratio of signal distribution inside and outside of the nuclear compartment. Thus, the exposure time was adjusted for each image to acquire a sufficient signal. The probe channel (Opal 570) exposure time was set at 100–200 ms for *ANO7* and *PPIB* and 250 ms for *dabB*. The confocal planes were collected at 0.27-µm intervals in the Z-dimension covering the full height of the sections.

### 4.5. Image Analysis

Maximum projection of image stacks was used for cropping everything except epithelium or cancer out of the images in ImageJ 1.53c (NIH, Bethesda, MD, USA), and the cropped stacks were imported into Imaris 8.1.2 (Oxford Instruments, Abingdon, UK). The nuclei were segmented as surfaces from the DAPI channel using the following parameters: smooth 0.5 μm (surface area detail level), background subtraction 2 µm (diameter of the largest sphere which fits into the objects), and the thresholding value was set based on visual evaluation in a range of 35–160. The resulting surfaces were filtered to include objects with a number of voxels greater than 5000, and a mask was created based on the segmented nuclei. The probe signal was segmented as spots using the following parameters: estimated XY diameter 0.5 µm with background subtraction, and the spots were classified with the automatic quality threshold value. In some negative control ROIs, the automated quality threshold value failed to segment the spots due to a low signal, and a manual threshold value was set. The spots were filtered based on the nucleus mask to result in a number of nuclear transcripts. The ratio of nuclear to total transcripts was calculated by dividing the number of nuclear spots by the total number of spots, resulting in a percentage of nuclear transcripts. The nuclei were counted manually.

### 4.6. Cell Culture

The MDA PCa 2b and 22Rv1 cell lines were acquired from the American Type Culture Collection (Manassas, VA, USA). MDA PCa 2b was grown in Ham’s F12 Kaighn’s modification (21127-022, Gibco, Waltham, MA, USA) supplemented with fetal bovine serum (FBS) (S1810, Biowest, Nuaille, France) to a final concentration of 20%, 25 ng/mL cholera toxin (C8052, Sigma-Aldrich, St. Louis, MO, USA), 0.005 mM phosphoethanolamine (P0503, Sigma-Aldrich, St. Louis, MO, USA), 100 pg/mL hydrocortisone (H0135, Sigma-Aldrich, St. Louis, MO, USA), 45 nM sodium selenite (9133, Sigma-Aldrich, St. Louis, MO, USA), 0.005 mg/mL human recombinant insulin (12585-014, Life Technologies, Waltham, MA, USA), 100 U/mL penicillin, and 0.1 mg/mL streptomycin. The cell line 22Rv1 was grown in RPMI-1640 (A10491, Gibco, Waltham, MA, USA) supplemented with 10% FBS, 100 U/mL penicillin, and 0.1 mg/mL streptomycin. Both cell lines were cultured at +37 °C in an atmosphere of 5% CO_2_ and 95% air.

### 4.7. Fractionation

The cells were cultured in a standard 10-cm dish to 50–90% confluency, detached with trypsin, and centrifuged at 125× *g* for 5 min at RT. The medium was discarded, and the cells were washed with PBS and centrifuged at 125× *g* for 5 min at RT. The PBS was discarded, and the cells were washed with ice-cold hypotonic buffer (10 mM HEPES pH 7.4, 1.5 mM MgCl_2_, 10 mM KCl, 1 mM EDTA, 0.5 mM DDT, 50 U/mL RNase inhibitor (N2611, Promega, Madison, WI, USA)) by dispersing the cells into 500 µL of the buffer. The samples were centrifuged at 125× *g* for 5 min at +4 °C, and the buffer was removed. Then, 500 µL of ice-cold hypotonic buffer was added to the cell pellet, and the cells were dispersed by pipetting followed by a 15-min incubation on ice. The cell suspension was gently passed through a 27-gauge needle 10 times avoiding foaming, and the cells were incubated on ice for an additional 20 min. The cells were dispersed by pipetting a few times, and 167 µL of suspension was transferred into a fresh tube for the whole cell sample. The remaining suspension was centrifuged at 720× *g* for 5 min at +4 °C. Then, 300 µL of supernatant for the cytoplasmic fraction was transferred into a fresh tube. The pellet containing the nuclear fraction was washed with 500 µL of hypotonic buffer with 0.15% (*v*/*v*) NP-40 and gently passed through a 25-gauge needle five times. The cytoplasmic and nuclear fractions were centrifuged at 720× *g* for 10 min at +4 °C. Then, 250 µL of cytoplasmic fraction supernatant was transferred into a fresh tube. The supernatant from the nuclear fraction was discarded, and the pellet was dispersed into 500 µL of hypotonic buffer with 0.15% NP-40. Both fractions were centrifuged again at 720× *g* for 10 min at +4 °C. Two hundred microliters of the cytoplasmic fraction supernatant was transferred into a fresh tube. The supernatant from the nuclear fraction was discarded, and the pellet was resuspended in 200 µL of hypotonic buffer. Next, 1800 µL of TRIsure (BIO-38032, Meridian Bioscience, Cincinnati, OH, USA) reagent was added to each sample of whole-cell lysate, cytoplasmic fraction, or nuclear fraction. The tubes were vortexed briefly and stored at −80 °C before RNA isolation. For protein samples, sodium dodecyl sulfate and cOmplete™ protease inhibitor cocktail (11836170001, Roche, Basel, Switzerland) were added to a final concentration of 1% and 1×, respectively.

### 4.8. Western Blot

Protein lysates in hypotonic buffer were sonicated (3-s pulses with 3-s breaks, 50% amplitude) with a Sonopuls ultrasonic homogenizer (Bandelin, Berlin, Germany) followed by protein concentration measurement with a Pierce™ BCA Protein Assay (23225, Thermo Fisher Scientific, Waltham, MA, USA). Protein lysate was mixed 1:1 with 2X Laemmli Sample Buffer (Bio-Rad Laboratories, Hercules, CA, USA) containing 5% 2-mercaptoethanol (Sigma-Aldrich, St. Louis, MO, USA), and 20 µg of protein was separated by polyacrylamide gel electrophoresis using Mini-PROTEAN^®^ TGX™ Precast Gels (Bio-Rad Laboratories, Hercules, CA, USA) and transferred into a 0.45-µm Immobilon^®^-P PVDF Membrane (Merck, Darmstadt, Germany). The membrane was stained with Ponceau S to assess the overall protein amount (Supplemental Figure S1). The membrane was blocked with 5% (*w*/*v*) nonfat dry milk in Tris-buffered saline with 0.1% Tween 20 (TBST) for 1 h at room temperature and incubated overnight on rotation at +4 °C in primary antibodies against HOXB13 (90944S, Cell Signaling Technology, Danvers, MA, USA) or beta-tubulin (sc-9104, Santa Cruz Biotechnology, Dallas, TX, USA) diluted 1:1000 in 5% *w*/*v* bovine serum albumin (BSA) in TBST. The secondary antibody (ab97080, Abcam, Cambridge, UK) was diluted 1:20,000 in 5% BSA-TBST and incubated for 1 h with rotation at room temperature. A WesternBright™ Quantum Western blotting detection kit (K-12042, Advansta, San Jose, CA, USA) was used for secondary antibody detection, and a ChemiDoc™ MP Imaging System (Bio-Rad Laboratories, Hercules, CA, USA) or LAS-4000 Luminescent Image Analyzer (Fujifilm, Tokyo, Japan) was used for imaging the membrane.

### 4.9. RNA Isolation

RNA was isolated from TRIsure lysates according to the manufacturer’s protocol with an additional chloroform phase separation step and two additional ethanol washes. The concentration of RNA was measured with a NanoDrop One (Thermo Fisher Scientific, Waltham, MA, USA), and the RNA integrity number (RIN) was examined with TapeStation 2200 (Agilent Technologies, Santa Clara, CA, USA) using a high-sensitivity RNA ScreenTape assay for analysis of total RNA. The RIN values were 8.2 or greater for total RNA, 7.7 or greater for cytoplasmic RNA, and 6.3 or greater for nuclear RNA.

### 4.10. ddPCR

Three biological replicates of samples were analyzed with ddPCR. RNA was treated with DNase I (18068015, Invitrogen, Waltham, MA, USA) according to the manufacturer’s protocol, and complementary DNA (cDNA) synthesis was performed with an iScript™ Select cDNA Synthesis Kit (1708897, Bio-Rad Laboratories, Hercules, CA, USA) using random primers. The reaction was incubated for 5 min at 25 °C, 30 min at 42 °C, and finally 5 min at 85 °C. The following assays were used in ddPCR: *ANO7* (Bio-Rad Laboratories assay ID dHsaCPE5026736 (FAM)) and *GAPDH* (Bio-Rad Laboratories assay ID dHsaCPE5031596 (FAM)). For *MALAT1*, the following primer sequences were designed and custom ordered from Bio-Rad Laboratories: forward primer 5′-GGTCTAGGATCCTCTACGCA-3′, reverse primer 5′-AAGAAGGAAGGAGCGCTAAC-3′, probe 5′-ACGCCTGCTCGCCTCCTCCGT-3′ (FAM). The amount of cDNA added to each reaction corresponded to the following amounts of RNA: *ANO7* 300 ng, *GAPDH* 6 ng, and *MALAT1* 0.6 ng. The reactions were set up according to the manufacturer’s protocol (22 µL/reaction) with ddPCR Supermix for Probes (no dUTP) (1863024, Bio-Rad Laboratories, Hercules, CA, USA) reagent. Droplets were generated with the Automated Droplet Generator (Bio-Rad Laboratories, Hercules, CA, USA). The following PCR program was run for all assays: step 1, 10 min at 95 °C; step 2, 30 s at 94 °C; step 3, 1 min at 57 °C; steps 2 and 3 were repeated 39 times; step 4, 10 min at 98 °C; and step 5, hold at 4 °C. The ramp rate at all steps was 2 °C/s. The plates were held overnight at 4 °C. On the following day, the droplets were read with a QX200 Droplet Reader (Bio-Rad Laboratories, Hercules, CA, USA). The raw data were analyzed with QX Manager Software 1.2 Standard Edition (Bio-Rad Laboratories, Hercules, CA, USA), and the results were normalized to copies per 1 µg of RNA.

### 4.11. Statistical Analyses

GraphPad Prism 8.4.2 (679) software (GraphPad Software, San Diego, CA, USA) was used to calculate the statistics. The group values were tested using the Shapiro–Wilk test for a normal distribution and the F test for equality of variances. An unpaired *t* test was used for the comparison of means with equal variances, and an unpaired *t* test with Welch’s correction was used for means with unequal variances. All *t* test *p* values are two-tailed, and statistical significance was defined at *p* < 0.05.

## Figures and Tables

**Table 1:** Nuclear enrichment of transcripts in prostate tissue.

	Nuclear %	SD	SEM	*n* Subjects ^1^	*n* ROIs	*n* Cells	*n* Spots ^2^
Benign glands and low-grade cancer							
*ANO7*	88.8	3.8	1.4	5	7	644	1163
*PPIB*	47.7	6.3	2.4	5	7	608	4337
*dabB*	67.4	20.7	9.3	4	5	280	53
High-grade cancer							
*ANO7*	77.7	9.3	3.5	5	7	817	975
*PPIB*	46.7	10.8	4.1	5	7	834	8985
*dabB*	57.8	23.7	10.6	4	5	437	228

^1^ Individual study subjects (patients) in the analysis. ^2^ Spots segmented from the confocal images.

**Table 2:** Transcript copy numbers in whole-cell, cytoplasmic and nuclear RNA.

	Cytoplasm		Nucleus		Whole Cell		Nuclear Enrichment
	Copies per 1 µg RNA	SD	Copies per 1 µg RNA	SD	Copies per 1 µg RNA	SD	Log2 Nucleus/Cytoplasm
22Rv1							
*ANO7*	117	30	1616	690	479	134	3.75
*MALAT1*	1.0 × 10^6^	0.4 × 10^6^	15.2 × 10^6^	2.9 × 10^6^	10.1 × 10^6^	5.6 × 10^6^	3.93
*GAPDH*	5.6 × 10^6^	1.8 × 10^6^	1.3 × 10^6^	0.4 × 10^6^	7.2 × 10^6^	2.3 × 10^6^	−2.07
MDA PCa 2b							
*ANO7*	3.6 × 10^3^	1.7 × 10^3^	22.0 × 10^3^	9.5 × 10^3^	6.5 × 10^3^	2.8 × 10^3^	2.63
*MALAT1*	9.4 × 10^6^	2.4 × 10^6^	102.7 × 10^6^	27.6 × 10^6^	30.1 × 10^6^	2.6 × 10^6^	3.44
*GAPDH*	41.8 × 10^5^	14.7 × 10^5^	7.3 × 10^5^	2.1 × 10^5^	25.5 × 10^5^	2.1 × 10^5^	−2.52

## Data Availability

The data presented in this study are available from the corresponding author upon reasonable request.

## Supplementary Materials

The following supporting information can be downloaded at: https://www.mdpi.com/article/10.3390/ijms24021052/s1.
