# Supplementary figures and images for: Transcripts of the Prostate Cancer-Associated Gene ANO7 Are Retained in the Nuclei of Prostatic Epithelial Cells
Source: Int J Mol Sci. 2023 Jan 5;24(2):1052. doi: 10.3390/ijms24021052 (PMC9865797; doi:10.3390/ijms24021052)

MDA PCa 2b

22Rv1

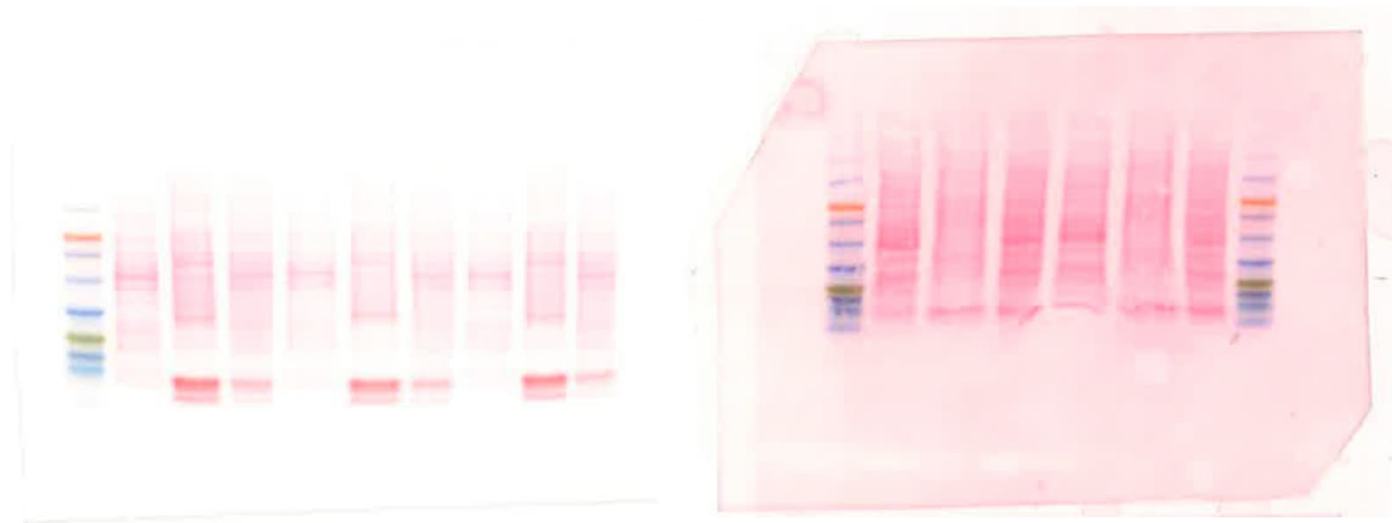

Supplement **Figure S1**. Ponceau S staining of the Western blot membranes.

Supplement: Supplementary file 1 [file ijms-24-01052-s001.zip › ijms-2115657-supplementary.pdf]
